# Beneficial Effects of Human Schwann Cell-Derived Exosomes in Mitigating Secondary Damage After Penetrating Ballistic-Like Brain Injury

**DOI:** 10.1089/neu.2023.0650

**Published:** 2024-11-15

**Authors:** Kengo Nishimura, Juliana Sanchez-Molano, Nadine Kerr, Yelena Pressman, Risset Silvera, Aisha Khan, Shyam Gajavelli, Helen M. Bramlett, W. Dalton Dietrich

**Affiliations:** ^1^ Department of Neurological Surgery, University of Miami Miller School of Medicine, Miami, Florida, USA.; ^2^ The Miami Project to Cure Paralysis, University of Miami Miller School of Medicine, Miami, Florida, USA.; ^3^ Interdisciplinary Stem Cell Institute, University of Miami Miller School of Medicine, Miami, Florida, USA.; ^4^ Neuroscience, Lacerta Therapeutics, Alachua, Florida, USA.; ^5^ Bruce W. Carter Department of Veterans Affairs Medical Center, Miami, Florida, USA.

**Keywords:** exosomes, human Schwann cells, inflammasome, inflammation, penetrating ballistic-like brain injury, traumatic brain injury

## Abstract

There is a growing body of evidence that the delivery of cell-derived exosomes normally involved in intracellular communication can reduce secondary injury mechanisms after brain and spinal cord injury and improve outcomes. Exosomes are nanometer-sized vesicles that are released by Schwann cells and may have neuroprotective effects by reducing post-traumatic inflammatory processes as well as promoting tissue healing and functional recovery. The purpose of this study was to evaluate the beneficial effects of human Schwann-cell exosomes (hSC-Exos) in a severe model of penetrating ballistic-like brain injury (PBBI) in rats and investigate effects on multiple outcomes. Human Schwann cell processing protocols followed Current Good Manufacturing Practices (cGMP) with exosome extraction and purification steps approved by the Food and Drug Administration for an expanded access single ALS patient Investigational New Drug. Anesthetized male Sprague-Dawley rats (280–350g) underwent PBBI surgery or Sham procedures and, starting 30 min after injury, received either a dose of hSC-Exos or phosphate-buffered saline through the jugular vein. At 48h after PBBI, flow cytometry analysis of cortical tissue revealed that hSC-Exos administration reduced the number of activated microglia and levels of caspase-1, a marker of inflammasome activation. Neuropathological analysis at 21 days showed that hSC-Exos treatment after PBBI significantly reduced overall contusion volume and decreased the frequency of Iba-1 positive activated and amoeboid microglia by immunocytochemical analysis. This study revealed that the systemic administration of hSC-Exos is neuroprotective in a model of severe TBI and reduces secondary inflammatory injury mechanisms and histopathological damage. The administration of hSC-Exos represents a clinically relevant cell-based therapy to limit the detrimental effects of neurotrauma or other progressive neurological injuries by impacting multiple pathophysiological events and promoting neurological recovery.

## Introduction

Traumatic brain injury (TBI) is a significant global cause of disability and death.^1,2^ In the United States, severe TBI is associated with thousands of deaths annually.^3^ Based on the latest report from the Centers for Disease Control and Prevention, the economic impact of TBI in 2010 was $76.5 billion. This includes both direct and indirect medical costs and represents the lifetime expenses associated with TBI. The majority of the cost, approximately 90%, is attributed to a fatal wound or hospitalization necessitated after TBI.^4^ A moderate or severe TBI not only affects long-term health problems and the lives of individuals and their families but also has an enormous societal and economic toll.^5^

Penetrating traumatic brain injury (pTBI) caused by bullets and shrapnel is a major concern in both civilian and military settings.^3,4^ Survivors often experience a high rate of disability as a result of pTBI, which is the most severe type of TBI and has the most dismal prognosis.^5,6^ Improvements in neuroimaging and advances in acute trauma management, however, have resulted in timely neurosurgical interventions and reduced gunshot deaths, leading to an increase in gunshot survivors. Unfortunately, there are no effective therapies available to aid in recovery beyond physical therapy, which can help reduce disability and cognitive deficits associated with TBI.^7,8^

Failure of regenerative neurogenesis, chronic inflammation, and atrophy after TBI lead to poor prognosis. Neuroinflammation can cause acute secondary injury after TBI and has been linked to chronic neurodegenerative diseases.^9,10^ Studies have shown that individuals who have a TBI experience a loss of gray and white matter over time resulting in brain atrophy. This phenomenon has been replicated in a rat model of penetrating ballistic-like brain injury (PBBI) where the loss of cells occurs rapidly, making acute neuroprotection alone insufficient in reducing damage ^11,12^

In a hospital setting, the primary objective is to minimize the impact of secondary injury, which can worsen the initial injury. Therefore, it is important to explore additional treatment options to address the long-term neuroinflammation associated with TBI and strategies to promote reparative processes.

Stem cell-based therapies have shown great potential in the management of different types of neurotrauma.^13–15^ The clinical application of stem cell-based therapies is challenging because of a lack of understanding of the migration, engraftment, and subsequent integration of transplanted stem cells into target brain circuits and concerns about the safety of stem cell therapy.^16^ Compared with stem cells, exosomes secreted from stem cells or other cell types may be a promising cell-free therapeutic option because of their unique properties, including stable biology and low immunogenicity.^17–19^

Exosomes are the smallest extracellular vesicles (30–150 nm) and are formed by the exocytosis of multi-vesicular bodies liberating vesicles on fusion with the cell's plasma membrane.^20,21^ They can contain various molecules such as lipids, proteins, and nucleic acids that are able to mediate long-distance communication from cell to cell.^22,23^ They can also exchange genetic information, transmit immune signals, and function as an independent metabolic unit.^19^ These vesicles may be capable of crossing the blood–brain barrier (BBB) and target sites of injury. Intravenous injection of several cell-derived exosomes has been shown to be safe and well tolerated and has the potential to regulate inflammation and restore neurological and motor functions.^24,25^

Microglia constantly monitor the brain environment to maintain normal brain function and homeostasis.^26^ Microglia are plastic and dynamically change in response to fluctuations in the brain environment and can activate and regulate neuroinflammation to either promote tissue repair or increase tissue damage, depending on phenotypic polarity.^27,28^ The continued development of systemically administrated therapies including exosome treatments focused on suppressing microglial-induced secondary tissue damage, and promoting tissue repair may be a promising strategy to reduce inflammation in TBI.

Recent studies have validated the efficacy of neural and mesenchymal stem cell (MSC)-derived exosomes in TBI animal models and demonstrated that exosomes exert their anti-inflammatory effects by modulating microglial function in several neurological diseases.^19,29,30^

In the peripheral nervous system, Schwann cells promote axonal dedifferentiation and proliferation after injury and remove myelin and axonal fragments.^31^ The regenerative abilities of Schwann cells have been applied to repair damage within the central nervous system (CNS).^32–34^ For example, the transplantation of human Schwann cells has been reported to provide tissue preservation after spinal cord injury (SCI) and support axon growth.^35,36^ Schwann cells that have undergone dedifferentiation secrete significant quantities of exosomes into the surrounding microenvironment after nerve injury and participate in the healing process.^37^ There have been a few studies using Schwann cell-derived exosomes after SCI, but there have been no reports on the effects of hSC-Exos in a TBI model.^38–40^

This is the initial report summarizing the consequences of intravenous administration of hSC-Exos into a rat model of pTBI, expanding on previous studies using the PBBI model.^41,42^ The study is considered novel in that hSC-Exos are utilized for the first time focusing on a severe model of TBI. Because of the importance of post-traumatic inflammatory processes in secondary injury, we focused on the effects of hSC-Exos treatment on inflammatory mediators, cell death mechanisms, and chronic histopathological damage. Based on these results, this highly translational exosome therapeutic approach has specific advantages that make it an exciting and innovative approach to the management of clinically relevant brain injuries.

## Methods

### Study design and animals

All experimental procedures were approved by the University of Miami's Animal Care and Use Committee and were conducted in compliance with the ARRIVE guidelines and those established by the National Institute of Health Guide for the Care and Use of Laboratory Animals.^43,44^ Animals were housed in a temperature-controlled room (22°C) and exposed to a 12h light/dark cycle. They were acclimated for at least seven days before surgery.

Male Sprague-Dawley rats (280–350 g) aged 2–3 months were used for this study. Male rats were chosen because males have been found to be more vulnerable to TBI than females, particularly during adolescence and young adulthood.^1^ In addition, hormonal fluctuations during the female reproductive cycle could introduce biological variability.

The study comprised four experimental groups including 10 rats per group for histological analysis and 5–6 rats for flow cytometry analysis in each group. These groups were labeled as Sham+phosphate-buffered saline (PBS), Sham+hSC-Exos, PBBI+PBS, and PBBI+hSC-Exos. To ensure unbiased results, the rats were randomly assigned to each group. To determine the appropriate sample size for the study, the researchers performed a power analysis based on previous histopathological and biochemical studies conducted in the PBBI model.^45-48^ This analysis helped to ensure that the study was adequately powered to produce meaningful and reliable results.^45,48–50^ Flow cytometry analysis was conducted 48h post-injury, and histology and immunocytochemistry at 21 days post-injury was analyzed.

### hSC-Exos isolation and characterization

Primary cultures of human Schwann cells were prepared and grown using methods similar to those used in several Food and Drug Administration (FDA) approved clinical trials (Investigational New Drug [IND] #14856 and 18909; NCT01739023, NCT02354625, NCT03999424) as described previously.^34,51,52^ All experiments were performed using banked cell cultures obtained from non-pathological nerve tissues that tested negative for blood-borne viruses. Cryopreservation of cell stocks was performed in a medium consisting of dimethyl sulfoxide (DMSO) and fetal bovine serum (FBS; Hyclone, GE Healthcare Life Sciences, South Logan, UT) at a ratio of 1:9.

Experiments used early passage Schwann cell cultures, usually collected after one to three rounds of subculture. Cryovials of human Schwann cells were thawed quickly at 37°C and resuspended in 10–15 mL of 1 × Dulbecco modified Eagle medium (DMEM; Life Technologies, Grand Island, NY) containing 10% FBS (heat-inactivated) before collection by centrifugation and plating directly onto a (1 μg/cm^2^) laminin-coated T-75 or T-150 flask at a density of 1–2 × 10^6^ cells/flask.

Cells were cultured in mitogens-supplemented growth media consisting of high-glucose DMEM, 10% FBS, 4 mM L-glutamine (Sigma-Aldrich, St. Louis, MO), 50 μg/mL of gentamicin, (APP Pharmaceutical/Fresenius Kabi USA, Lake Zurich, IL), 10 nM heregulin (Genentech, South San Francisco, CA), and 2 μM forskolin (Sigma-Aldrich, St. Louis, MO). The cells were maintained in a 37°C incubator set to 8–9% CO_2_ for optimal growth. The cultures were observed under a phase contrast microscope to confirm cell adhesion to the substrate within 2–3h of seeding. Regular media changes were performed until the cells reached confluency (70–80% confluence).

At this point, regular media were removed from Schwann cells, cells were washed two times with PBS to remove any remaining FBS, replaced by FBS free medium, and cultured for 48h. The hSC-Exos were then isolated from the supernatant of Schwann cells. Exosome extraction and purification steps were also reviewed and approved by the FDA for an expanded access single ALS patient IND application #28561. Briefly, the supernatant was collected and centrifugated at 3000 *g* for 10 min to remove cell debris and filtered through a 0.2 μm filter, and then subjected to centrifugation under 100,000 *g* for 130 min at 4°C. The supernatant was then aspirated, and the pellet was resuspended into 500 μL of cold PBS, then aliquoted and stored at -80°C.

### hSC-Exos characterization

**FIG. 1.**
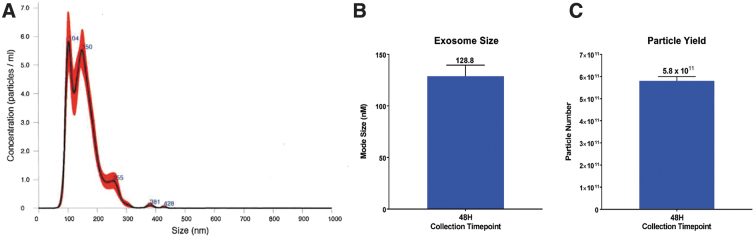
Nanosight analysis of hSC-derived exosomes. (**A**) Nanosight nanoparticle analysis of hSC-derived exosomes. (**B**) Graph demonstrating hSC-derived exosome size. (**C**) Graph representing the hSC-derived exosome concentration.

**FIG. 2.**
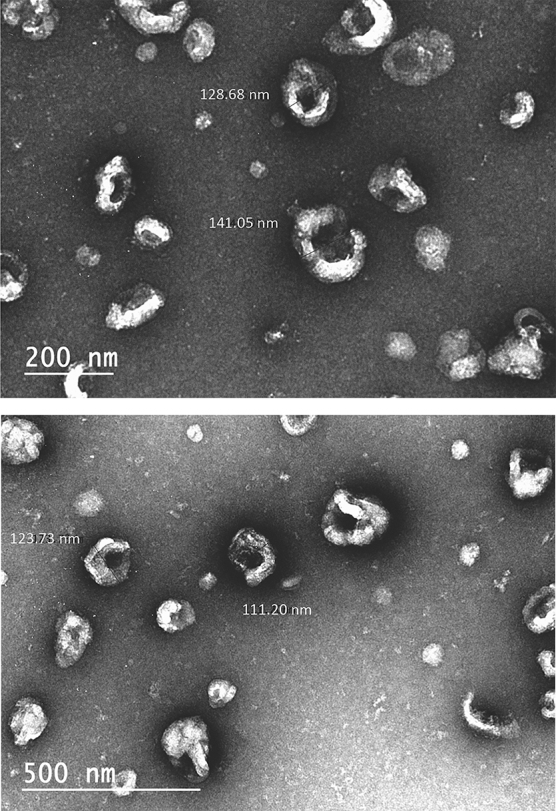
Transmission electron microcopy (TEM) of hSC-derived exosomes. hSCs were isolated and imaged using TEM.

The size distribution and concentration of hSC-Exos in a 1 mL solution were visualized and quantified using the NanoSight NS300 (Malvern Instruments Ltd.) and NTA 3.3 software (Fig. 1). Nanosight Tracking Analysis confirmed the mean size of hSC-Exos was 120.9 ± 3.3 nm, standard deviation (SD) was 59.1 ± 5.1 nm, and concentration was 5.4** ×** 10^11^ ± 3.5** ×** 10^10^ particles/mL. Further, hSC-Exos were subjected to transmission electron microscopy (TEM) to confirm morphology and size. Total protein concentration was measured using DC Protein Assay, following a Microplate Assay Protocol (0.340 mg/mL). hSC-Exos were positive for cell membrane markers: CD63 (95.9%), CD81 (95.9%), and CD9 (94.8%) by flow cytometry. TEM analysis revealed that hSC-Exos were round or elliptical vesicles with diameters of approximately 30–150 nm (Fig. 2).

### PBBI surgery

PBBI surgery was conducted as described previously.^40,53^ Surgical procedures were performed under aseptic conditions. Rats were anesthetized with 2–5% isoflurane delivered in a mixture of 70% nitrous oxide and 30% oxygen. A homoeothermic heating pad was used to maintain the rat's body temperature at 37 ± 1°C while a feedback heat lamp system kept the head temperature at the same level. An indwelling catheter (PE-50) tubing with a 2.9 cm Silastic tubing leader was surgically implanted into the right jugular vein before the PBBI surgery for intravenous delivery of hSC-Exos.

The rat's head was secured in a stereotaxic frame (Kopf, Tujunga, CA) to insert the PBBI probe (Kadence Science, Lake Success, NY), which is a 20G stainless steel tube with fixed perforations along one end, sealed by a piece of airtight elastic tubing. The unperforated end was attached to a hydraulic pressure-pulse generator (4B080; MITRE, MA) angled at 50 degrees from the vertical axis and 25 degrees counterclockwise from the midline. A midline scalp incision was made, and a right frontal cranial window (diameter 4 mm) was created using a drill to expose the right frontal pole (+4.5 mm anterior-posterior, +2 mm medial-lateral to bregma).^54^

The PBBI probe was advanced through the cranial window into the right hemisphere to a depth of 12 mm from the brain's surface. Once the probe was in place, a pressure pulse was released by a computer, calibrated to produce a rapid expansion of the water-filled elastic tubing, causing an elliptical-shaped balloon (diameter = 0.633 mm, duration = 40 msec) to form. This created a temporary unilateral cavity in the brain, mimicking the generation of a ballistic force shock wave. After the deflation, the probe was removed from the brain, and the skin incision was closed with wound clips.

Sham surgeries consisted of the midline scalp incision, the right frontal burr hole, and the skin incision was closed with wound clips. Rats were randomly given PBS or a dose of 300 μL (1 × 10^11^ particles) hSC-Exos through the jugular vein 30 min after PBBI or Sham operation. This dose was based on preliminary studies in the laboratory.

### Flow cytometry

To analyze the phenotype through flow cytometry, a modified version of a recent method was used.^40,55^ After 48h of injury, animals were anesthetized with 2-5% isoflurane and then perfused with cold PBS for 6 min. The ipsilateral cerebral cortex was dissected on ice and placed in ice-cold Hank Balanced Salt Solution. Neural tissue was dissociated into single-cell suspension by enzymatic digestion using the Neural Tissue Dissociation Kit (Miltenyi Biotec) and mechanical dissociation by passing through a 70-μm cell strainer (Falcon, Madison, WI). Debris and myelin were removed using Debris Removal Solution (Miltenyi Biotec).

Cells were labeled for caspase-1 activity with a FAM-FLICA assay (Immunochemistry Technologies, Bloomington, MN) following the manufacturer's instructions. Cell viability was determined using LIVE/DEAD Fixable Blue Dead Cell Stain Kit (Invitrogen). CD32 (BD Biosciences) antibodies were used for a non-specific block, and then cells were labeled with surface markers such as CD11b (BD Biosciences) and CD45 (BD Biosciences). Cells were then fixed with BD Cytofix (BD Biosciences).

Cell populations were gated to include only live cells, and standard forward and side scatter gating was used in the analysis. CD45 and CD11b were used to distinguish between CD45_low_ resting microglia (CD45_low_, CD11b+), CD45_intermediate_ activated microglia (CD45_int_, CD11b+), and CD45_high_ infiltrating myeloid-lineage cells (CD45_high_, CD11b+). Activated microglia increase their expression of CD45 compared with surveying microglia while infiltrating leukocytes, including macrophages, monocytes, and neutrophils, express the highest amounts of CD45. We established appropriate gates by using the antibody isotype controls provided by the manufacturer and by running a single-color control for each marker.

To acquire the samples, we used Beckman Coulter CytoFLEX S with CytExpert 2.0 as acquisition software. The resulting FCS files were analyzed using Kaluza 1.5A software provided by Beckman Coulter.

### Histopathology and immunohistochemistry (IHC)

After 21 days of either PBBI or Sham procedures, the animals were anesthetized with 3% Isoflurane in a mixture of 30% O_2_ and 70% N_2_O. They were then transcardially perfused with saline for 1 min, followed by 0.1 M phosphate buffer (pH 7.4) containing 4% paraformaldehyde at a pressure of 100–120 mm Hg for 15 min, all at room temperature. This was performed by a surgeon who was unaware of the animal's condition in respect to PBBI or Sham procedures.

Next, the brain tissues were carefully removed from the skull and post-fixed in the same fixative for 6h at 4°C. After this, the brains were transferred into 0.1 M phosphate buffer (pH 7.4) containing 20% sucrose and stored at 4°C. The sucrose solution was replaced after the first 6h of immersion. Finally, the brains were sent to FD NeuroTechnologies (Columbia, MD) for histopathological and immunostaining procedures.

To analyze brain injury, the cerebrum was cut into sections 30 μm thick, coronally from 6.12 to -9.12 mm anteroposterior to the bregma. These sections were collected at 300 μm intervals and processed in two groups. One group was stained with hematoxylin and eosin (H&E) using H&E solutions (FD NeuroTechnologies) to evaluate the injury's morphology. The other group was processed for Iba-1 (ionized calcium binding adaptor molecule-1) IHC using anti-Iba-1 (FUJIFILM Wako Chemicals U.S.A. Corporation).

Digital imaging techniques were utilized to capture H&E-stained brain tissues from a total of 20 coronal brain sections, each spaced 300 μm apart, ranging from +4.92 mm to -6.48 mm from bregma. Volume quantification was achieved through the utilization of contouring with Neurolucida (version 7.50.1, MicroBrightField Inc., Williston, ME), with the Neuroexplorer program employed for volume calculations.^56,57^ Volumes were determined by tracing the boundaries of each structure at a power of 10x using a camera lucida microscope attachment. To determine the differences in tissue shrinkage, percent atrophy was determined by calculating the difference between ipsilateral and contralateral volume and normalizing to the contralateral volume.

Our analysis focused specifically on several distinct regions, including the isocortex, corpus callosum, hippocampal region (Ammon horn, dentate gyrus, subiculum), lateral ventricles (inclusive of porencephalic cysts), lesion cavity, and perilesional region (injured penumbra and degeneration region). The injured region was defined as the lesion cavity in addition to the injured penumbra and degeneration region, which encompassed neuropil pallor and rarefaction, neuronal necrosis and loss, disruption of neuropil architecture, and gross hemorrhage.

### Stereological counts of microglia subtypes

An unbiased investigator assessed the stereological counts of microglial activation patterns on immunostained Iba-1 serial sections using StereoInvestigator (MBF Bioscience, Williston, VT). A counting frame of 75** ×** 75 μm and a counting grid of 500** ×** 500 μm were employed by the investigator to determine the number of microglia present in the ipsilateral cortex of Sham-operated animals and the contralateral and ipsilateral cortices of PBBI animals (N = 5–6 per group). The number of microglia was classified into four distinct activation states based on their morphology, as outlined by Torres-Platas and colleagues.^58^

### Statistical analysis

All statistical analysis procedures were blindly conducted on GraphPad Prism 9.5.0 (GraphPad Software, Inc., La Jolla, CA). Data are expressed as mean ± standard error of the mean (SEM) with *p* < 0.05 considered significant in all statistical tests. Stereological results (microglia counts) and flow cytometry results were compared using one-way analysis of variance (ANOVA) followed by the Tukey multiple comparisons test. We performed a repeated measures ANOVA for the lesion area across bregma levels, the Wilcoxon match-pairs signed rank test for the cavity + perilesional and a one-way ANOVA for group comparisons of ipsilateral cortical volume followed by the Tukey multiple comparison test.

## Results

### Delivery of hSC-Exos decreases lesion volume after PBBI

**FIG. 3.**
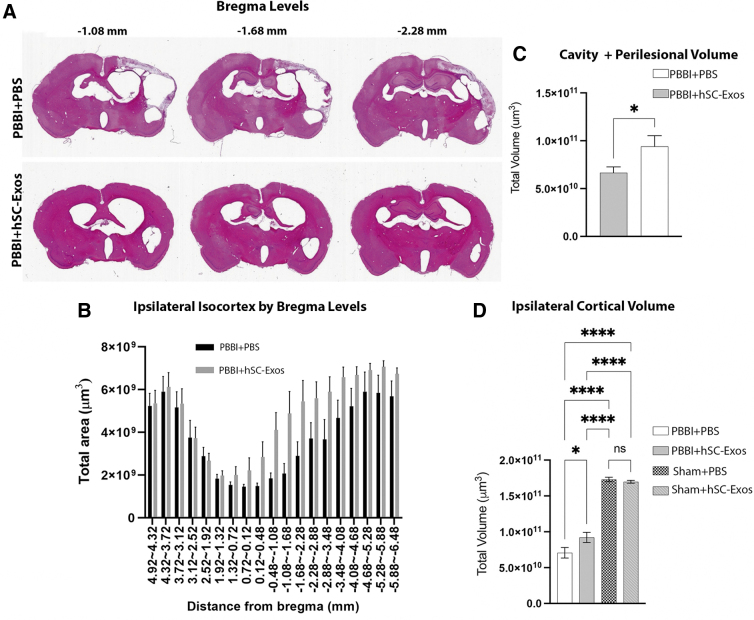
Lesion volume analysis 21 days after penetrating ballistic-like brain injury (PBBI) and hSC-Exos treatment. (**A**) Representative images showing that hSC-Exos treatment significantly decreases lesion volume 21 days after PBBI between bregma levels -1.08 -1.68. (**B**) Quantification of lesion area analysis by bregma level using a two-way repeated measures analysis of variance (ANOVA) (*n* = 10/group). Group comparison was significant (*p* < 0.05), bregma levels were significant (*p* < 0.05) but group** ×** bregma was not significant (*p* = 0.3112). The significance of the group and bregma level changes indicate that overall there are differences between treatment groups as well as changes in the lesion area throughout the neuraxis. (**C**) PBBI + hSC-Exos treated rats had significantly decreased cavity + perilesional volume compared with PBBI+phosphate-buffered saline (PBS) treated rats at 21 days post-PBBI (*n* = 10, *p < 0.05) using a Wilcoxon match-pairs signed rank test. (**D**) One-way ANOVA showed a significant difference in ipsilateral cortical volume between groups (*p* < 0.0001). *Post-hoc* analysis showed that PBBI+hSC-Exos treated rats demonstrated a statistically significant increase in ipsilateral lesion volume 21 days post-PBBI compared with PBBI + PBS treated rats (*p < 0.05). Additional group comparisons were significant for PBBI+PBS and Sham+PBS (****p < 0.0001), PBBI+PBS and Sham+hSC-Exos (****p < 0.0001), PBBI+hSC-Exos and Sham+PBS (****p < 0.0001), PBBI+hSC-Exos and Sham+hSC-Exos. (****p < 0.0001). (*n* = 10/group).

One of the characteristics of the PBBI model is that it is designed to reproduce the temporary cavity that forms with high-speed penetration as seen in the penetrating TBI population.^59^ The lesion cavity plus injured penumbral regions again defined by neuropil pallor, neuronal necrosis, disruption of neuropil architecture, and regions of gross hemorrhage were included.^54^ Our results showed that treatment with hSC-Exos 30 min after PBBI significantly (*p* < 0.05) decreased lesion cavity + perilesional volume at 21 days post-injury (Fig. 3C).

When examining lesion areas by bregma levels, we found that there was a significant difference between groups (*p* < 0.05), bregma levels (*p* < 0.05) but no interaction (*p* = 0.3112). These data indicate that there is an overall group difference between PBBI + hSC-Exos treated rats compared with the PBBI + PBS treated rats demonstrating a positive improvement in lesion area for exosome treatment (Fig. 3B). Representative images are also shown to demonstrate the lesion area changes across three bregma levels (Fig. 3A).

Last, we also demonstrated that the administration of hSC-Exos 30 min after PBBI significantly (*p* < 0.0001) increased ipsilateral cortical volumes at 21 days post-injury (Fig. 3D). These results indicate that treatment with hSC-Exos has neuroprotective properties that may be beneficial to recovery of tissue loss because of cavity formation and preservation of penumbral regions after pTBI.

### Delivery of hSC-Exos reduces number of activated microglia 48h after PBBI

**FIG. 4.**
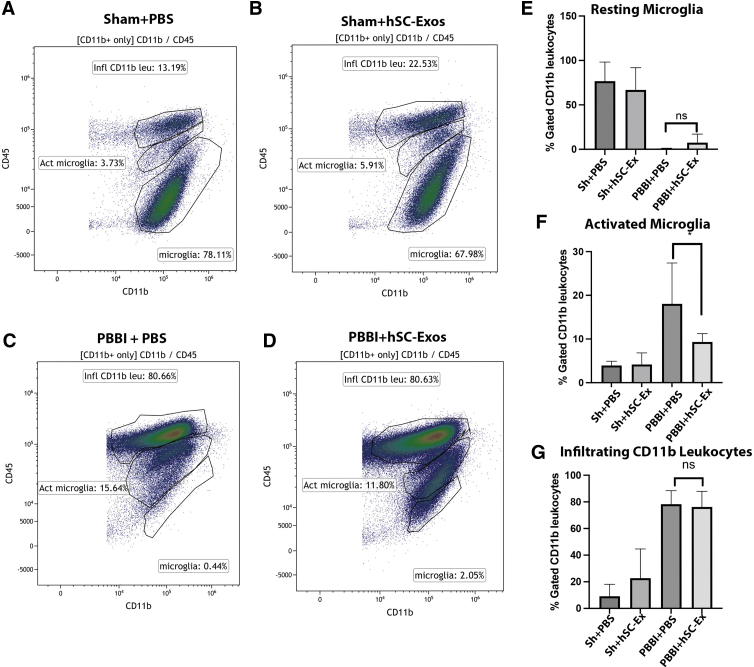
Flow cytometry analysis of activated microglia 48h after penetrating ballistic-like brain injury (PBBI) and treatment with hSC-Exos. Representative flow cytometry scatter density plots from cortical tissue of (**A**) Sham + phosphate-buffered saline (PBS), (**B**) Sham + hSC-Exos, (**C**) PBBI + PBS, (**D**) PBBI + hSC-Exos 48h after PBBI. Quantification of (**E**) resting microglia, (**F**) activated microglia, (**G**) infiltrating CD11b leukocytes. The PBBI + hSC-Exos (PBBI+ hsC-Ex) treatment 48h after PBBI significantly decreased the number of activated microglia (F). There was no significant difference in the number of resting microglia (E) or infiltrating CD11b positive leukocytes (G). Statistical significance was determined using a one-way analysis of variance, *n* = 5–6/group, *p < 0.05, ns = no significance.

Flow cytometry analysis of cortical tissue at the site of the lesion was performed to differentiate resting microglia, activated microglia, and infiltrating immune cells, such as macrophages and neutrophils.^40,55^ As reported in our previous studies, vehicle treated PBBI rats consistently show increased expression of activated microglia and leukocytes and lower levels of resident microglia compared with Sham injured mice 48h after injury (Fig. 4).^40^ This was illustrated by the tight cluster of CD11+ and CD45_high_ cells (Fig. 4C) compared with the tight cluster of CD45_low_ and CD11b+ cells in the Sham rats (Fig. 4A). Rats treated with hSC-Exos 48h after PBBI (PBBI+hSC-Exos) had significantly (*p* < 0.05) decreased numbers of activated microglia compared with PBBI-vehicle (PBBI + PBS) treated rats (Fig. 4F). This is also demonstrated by the tighter cluster of activated microglia (CD45_int_ and CD11b+) in the representative scatter density plots (Fig. 4 C,D).

Although there was a slight increase in resting microglia in PBBI+ hSC-Exos compared with PBBI+PBS, there was no statistical significance (Fig. 4E). In addition, there was no significant change in the number of infiltrating CD11b leukocytes between both groups (Fig. 4G). These results suggest that treatment with hSC-Exos after PBBI has potential anti-inflammatory effects at 48h after injury by reducing the number of activated microglia.

### Delivery of hSC-Exos reduces caspase-1 expression in activated microglia 48h after PBBI

Our previously published data showed that inflammasome activation and pyroptotic cell death increased 48h after PBBI.^53^ In the present study, FAM-FLICA and a LIVE/DEAD assay were used to determine cell-viability. FAM-FLICA is a fluorescent probe against amine residues of protein and permeates the cell membrane.^60^ When cell death occurs, such as pyroptosis, the LIVE/DEAD, which is membrane impermeable at a resting state, enters the cell and increases the fluorescence of cells.

**FIG. 5.**
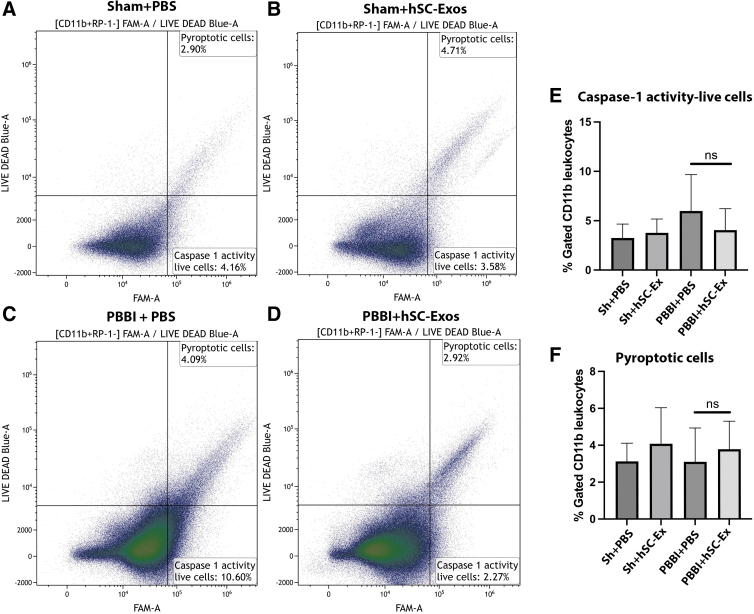
Flow cytometry analysis of caspase-1 activation 48h after penetrating ballistic-like brain injury (PBBI) and treatment with hSC-Exos. Representative flow cytometry scatter density plots of caspase-1 activity (FAM-FLICA) versus LIVE/DEAD from cortical tissue of (**A**) Sham + phosphate-buffered saline (PBS), (**B**) Sham + hSC-Exos, (**C**) PBBI + PBS, (**D**) PBBI + hSC-Exos 48h after PBBI. CD11b positive cells were gated based on high FLICA expression and high LIVE/DEAD expression for pyroptotic cells. Caspase-1 activity in live cells was determined by high FLICA expression and low LIVE/DEAD expression. Quantification of (**E**) caspase-1 activity live cells and (**F**) pyropotic cells. There was no significant difference in the number of caspase-1 activity in live cells or cells undergoing pyroptosis. Statistical significance was determined using a one-way analysis of variance, *n* = 5–6, ns = no significance.

The following gates were established to determine caspase-1 activity in all CD11b+ with the exclusion of RP1+ cells after hSC-Exos treatment 48h post-PBBI (Fig. 5), live CD11b+ cells that do not express caspase-1 activity (FLICA_low_, LIVE/DEAD_low_), live CD11b+ cells that express caspase-1 activity (FLICA_high_, LIVE/DEAD_low_), and pyroptotic CD11b+ cells (FLICA_high_, LIVE/DEAD_high_).^40^

Although there was a decrease in caspase-1 activity in live cells after hSC-Exos treatment 48h post-PBBI, the results were not significant. (Fig. 5E). Further, there was no change in the number of pyroptotic cells with hSC-Exos treatment 48h after PBBI (Fig. 5F).

**FIG. 6.**
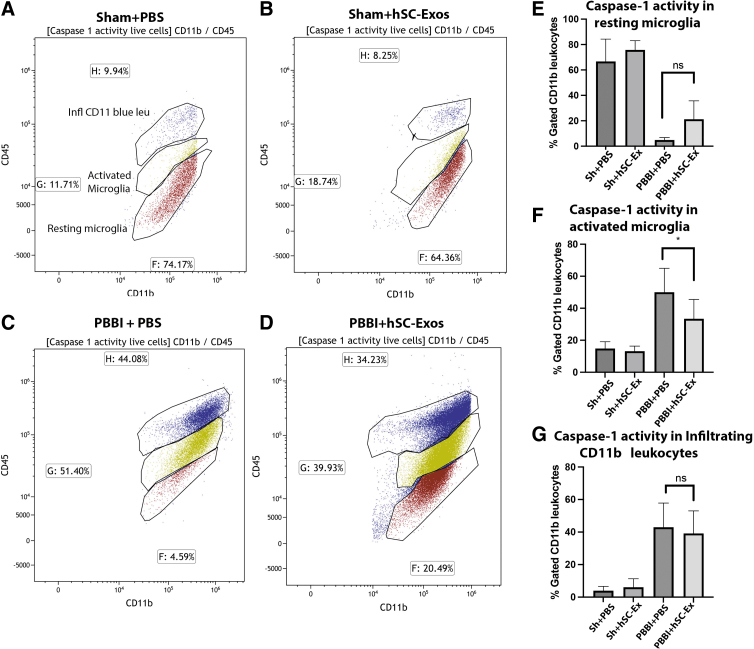
Flow cytometry analysis of caspase-1 activation in activated microglia and infiltrating leukocytes 48h after penetrating ballistic-like brain injury (PBBI) and treatment with hSC-Exos. Representative flow cytometry scatter density plots of CD11b+ leukocytes, activated microglia, and resting microglia that have caspase-1 activity or are pyroptotic cells of (**A**) Sham + phosphate-buffered saline (PBS), (**B**) Sham + hSC-Exos, (**C**) PBBI + PBS, (**D**) PBBI + hSC-Exos 48h after PBBI. Cells that were gated for caspase-1 activity in live cells or pyroptotic cells were further gated into the three groups mentioned above. Quantification of (**E**) resting microglia, (**F**) activated microglia, (**G**) infiltrating CD11b leukocytes that are either caspase-1 active or undergoing pyroptosis. The PBBI + hSC-Exos (PBBI+ hsC-Ex) treatment 48h after PBBI significantly decreased the number of activated microglia (F). There was no significant difference in the number of resting microglia (E) or infiltrating CD11b positive leukocytes (G). Statistical significance was determined using a one-way analysis of variance, *n* = 5-6, *p < 0.05, ns = no significance.

The following gates were next used to determine whether hSC-Exos treatment reduces caspase-1 activity and pyroptosis in activated microglia and infiltrating leukocytes: (FLICA_high_, LIVE/DEAD_high_) for pyroptois and (FLICA_high_, LIVE/DEAD_low_) for live caspase-1 activity. In addition, the CD11b+ cells were further gated into resting microglia (CD45_low_, CD11b+), activated microglia (CD45_int_, CD11b+), and infiltrating leukocytes (CD45_high_, CD11b+) as shown in Figure 6.

Although there was no significant difference in the reduction of caspase-1 activity in all CD11b+ cells after hSC-Exos treatment, there was a significant (*p* < 0.05) reduction of caspase-1 activity in activated microglia 48h post-PBBI treated animals (Fig. 6F). This was demonstrated in the representative density scatter plots where there is the presence of a tighter cluster of cells that are CD45_int_, CD11b+ with caspase-1 activity in the PBBI+PBS group compared with the PBBI+exos group (Fig. 6C,D). In addition, both Sham+PBS and Sham+hSC-Exos groups (Fig. 6A,B) portrayed lighter clusters of CD45_int_, CD11b+ compared with PBBI+PBS and PBBI+hSC-Exos groups (Fig. 6C,D).

Last, there was also a mild increase in the caspase-1 activity in the resting microglia (CD45_low_, CD11b+) (Fig. 6E). These results suggest that hSC-Exos treatment 48h after PBBI may have an anti-inflammatory effect through the inflammasome pathway in activated microglia.

### Delivery of hSC-Exos after PBBI reduces number of Iba-1 activated and amoeboid microglia

**FIG. 7.**
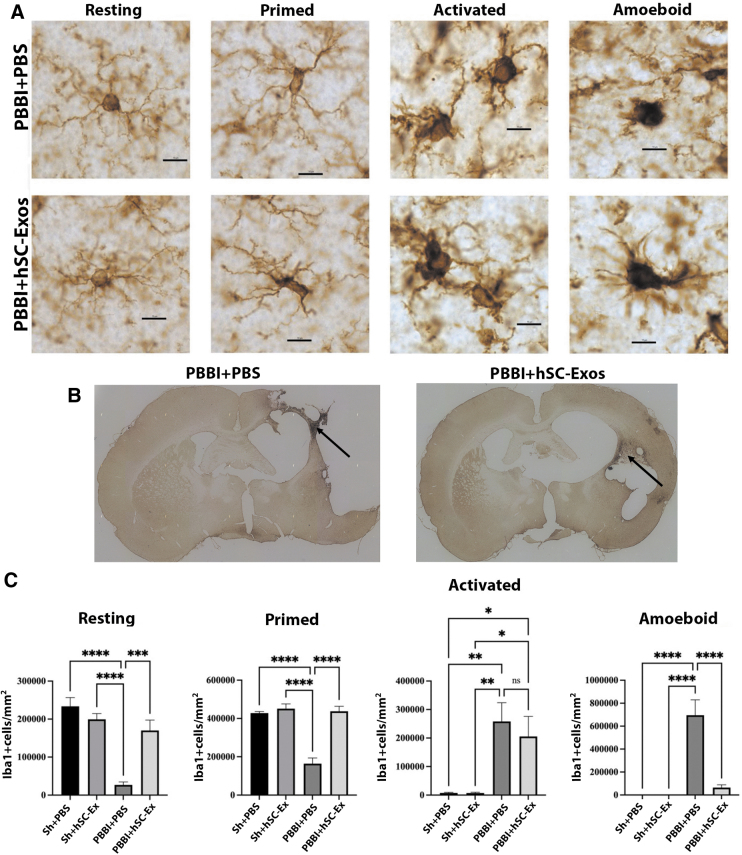
hSC-Exos treatment reduces the number of Iba-1 positive activated and amoeboid microglia 21 days after penetrating ballistic-like brain injury (PBBI). (**A**) Representative images (60x) of microglia that were used for unbiased stereological counts based on morphology. Cells were classified into four major types: resting, primed, activated, and amoeboid. The scale bars represent 10 μm. (**B**) Representative lower magnification images (10x) in the injured cortex where higher magnification images were taken. Black arrows point to the perilesion where images were taken. (**C**) Quantification of Iba-1+ microglia in the ipsilateral cortex. There was an increase in activated and amoeboid cells after PBBI compared with sham-operated groups. There was a significant (*p* < 0.05) increase in resting and primed microglia and a significant (*p* < 0.05) reduction in amoeboid in the PBBI group administrated with hSC-Exos. Data are presented as mean ± standard error of the mean. Statistical significance was determined with one-way analysis of variance followed by the Tukey multiple comparison test. *p < 0.05, **p < 0.01, ***p < 0.001, ****p < 0.0001. *n* = 5–6 per group.

Previous studies have shown that PBBI increases the number of activated microglia compared with Sham operated mice up to 12 weeks post-injury.^40,53^ In this study, we report the effect of hSC-Exos treatment on microglial morphological changes three weeks after PBBI using Iba-1 immunocytochemistry (Fig. 7). Results show that treatment with hSC-Exos 30 min after PBBI significantly (*p* < 0.05) increases the number of resting and primed microglia (Fig. 7C). Supporting our previous studies where we also found that the number of activated microglia are significantly (*p* < 0.05) increased after PBBI compared with Sham operated rats three weeks after PBBI (Fig. 7C).^40,53^

Although our results did not show a significant decrease in the number of activated microglia in the PBBI + hSC-Exos group compared with the PBBI + PBS group, we did find that the PBBI + hSC-Exos treatment significantly (*p* < 0.05) reduced the number of amoeboid microglia compared with PBBI + PBS treatment at three weeks after PBBI (Fig. 7C).

**FIG. 8.**
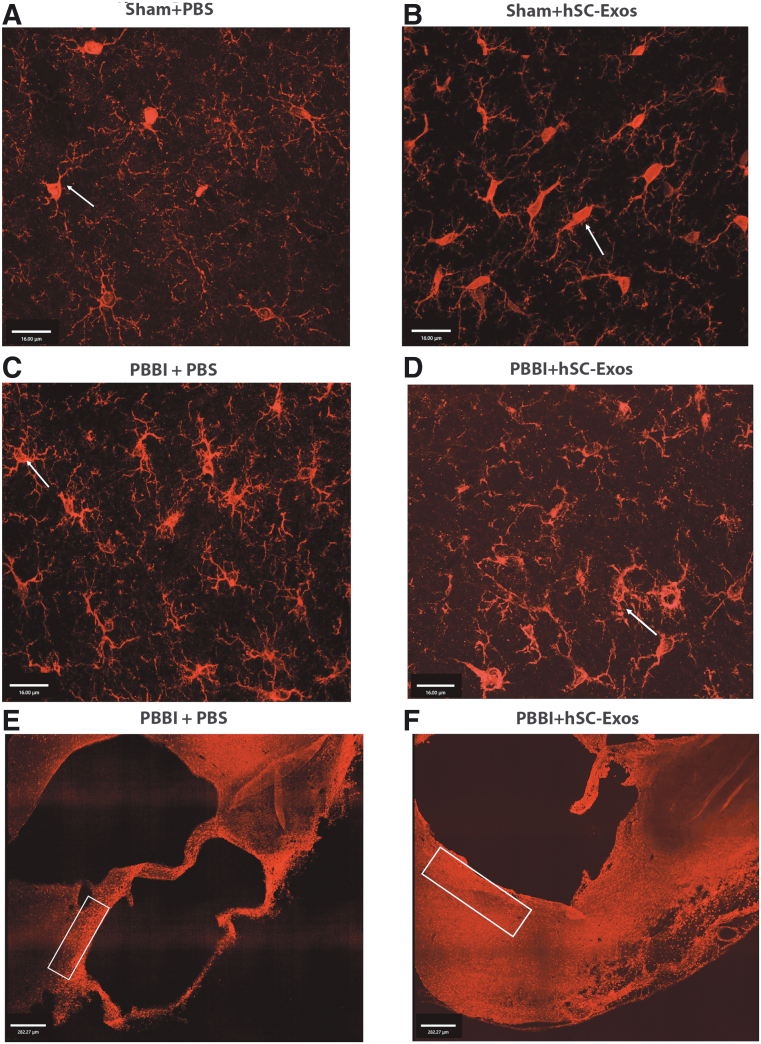
Immunohistochemical representation of Iba-1 positive microglial morphology changes after penetrating ballistic-like brain injury (PBBI) and hSC-Exos treatment. (**A**) Sham + phosphate-buffered saline (PBS) and (**B**) Sham + hSC-Exos treatment rats have increased presence of resting microglia compared with injured groups (Iba1-red). White arrows indicate resting state microglia. (**C**) PBBI + PBS treated rats have increased activated microglia as demonstrated by white arrow. (**D**) PBBI + hSC-Exos treatment rats have less activated microglia compared with the PBBI+PBS group as well as increased primed microglia (white arrow) (Iba1-red) in A–D images taken at 60x; scale bar = 16 μm. (**E,F**) Representative 10x images of perilesional area of injured cortex where higher magnification images were taken (white box); scale bar = 282.27 μm.

For the quantification of morphological microglial changes, cell counts were taken from the perilesional area of the injured cortex (Fig. 7B- black arrows). We also performed fluorescent IHC staining to further examine the neuroprotective effect of hSC-Exos on microglial morphology at three weeks after PBBI (Fig. 8). As demonstrated by the white arrows in Figure 8, PBBI + hSC-Exos (Fig. 8D), treated rats show an increased presence of resting and primed microglia while PBBI + PBS treatment demonstrated an abundance of amoeboid microglia (Fig. 8C).

As demonstrated in Figure 8 E and F, activated microglia were most prominent around the perilesional area of the injured cortex (white box) where higher magnification images were taken. These results further confirm our microglial cell counting (Fig. 7) and provide evidence that hSC-Exos treatment ameliorates microglial morphology at a chronic time point after PBBI.

## Discussion

The incidence of pTBI is increasing in the United States, particularly in young individuals.^61^ One of the components of pTBI is the formation of a cavity that occurs after the trauma, which has been successfully modeled in the PBBI model used in this study. Penetrating brain damage is both focal and diffuse characterized by tissue disruption, contusion hematoma, and associated with widespread delayed neuronal loss because of pressure and shearing forces causing cavitation.^62,63^ Secondary injury mechanisms, such as programmed cell death and detrimental inflammatory responses, are potential therapeutic targets for promoting neurological repair and improving functional outcomes. In this current study, we used the well-established PBBI model to examine for the first-time whether hSC-Exos treatment could target secondary injury mechanisms and reduce histopathological damage.

In recent years, exosomes have been increasingly studied for their importance in cell-to-cell communication as well as having the potential to provide new therapeutic strategies for various conditions, including neurotrauma.^64,65^ There are currently more than 150 clinical trials examining exosomes as a form of therapeutic intervention for a variety of human diseases.^66^

Exosomes are known to participate in important cellular activities involving the immune and inflammatory response, apoptosis, and angiogenesis.^19,67–70^ Exosomes from various cell types including neural stem cells have been reported to be promising therapeutic agents for the management of acute CNS injury.^64,71,72^ Also, evidence shows that molecules such as siRNAs, miRNAs, and proteins can be loaded into exosomes and delivered to specifically targeted cells.^73,74^

Exosomes are membrane bound vesicles of endosomal origin that are secreted by most cell types and internalized mainly because of endocytosis with other cells to deliver their intracellular contents.^72^ In the field of TBI research, exosomes have become a promising alternative to other cell-based therapies, such as stem-cell therapy, primarily because they can be administered intravenously and have low immunogenicity.

There have been studies on MSC-derived exosomes therapy after TBI, with one recent study demonstrating that treatment with MSC-derived exosomes after a controlled cortical impact injury reduced pyroptosis, a form of inflammasome-induced cell death.^75^ In another study, bone marrow stromal cells-derived exosomes enriched with miR-124-3p alleviated glutamate-mediated excitotoxity and improved neurological recovery after TBI.^76^

Astrocyte-derived exosomes transduced with the connexin-43 gap junctional protein have also been reported to improved neurological outcomes after TBI. ^77^ The current study builds on recent studies where exosome therapy was tested and for the first time evaluated human Schwann cell-derived exosomes in a severe TBI model.

Schwann cells produce chemokines and cytokines that are important for macrophage recruitment and myelin removal during Wallerian degeneration and ensuring axonal regeneration.^33,78,79^ hSC-Exos have been implicated as potential therapeutic interventions in peripheral nerve injury and SCI.^32,80^ Recently, studies have investigated the potential application of rodent SC-Exos to deliver therapeutics and function as surrogate biomarkers.^31,39,81,82^ Rodent SC-Exos enhance regeneration of the peripheral nervous system and may improve diabetic peripheral neuropathy in a Type 2 diabetic mouse model. ^37,82^

In a recent SCI study, Huang and colleagues^32^ reported that rodent SC-Exos attenuate tissue damage and improve functional recovery by promoting angiogenesis. Also, rodent SC-Exos have been reported to promote recovery after SCI by increasing axonal protection by increasing autophagy and decreasing apoptosis ^39^

Recent studies have highlighted the emerging role of exosomes in communication between the peripheral and the CNS.^24^ Although exosomes have been reported to be capable of crossing the BBB under specific conditions, transport mechanisms across vascular and other barriers remain to be fully characterized.^24,25,83–86^ The release of exosomes from various CNS cell types regulates BBB-associated phenomena including tumor dynamics, angiogenesis, and immune responses.^87–89^ Specific transport mechanisms for exosomes through the BBB include endocytosis, micropinocytosis, phagocytosis, and plasma membrane fusion.^90,91^

In this study, hSC-Exos injected into the jugular vein after PBBI may have therefore crossed both intact as well as trauma-induced permeable vascular beds leading to the release and uptake of exosome cargo into various cell types of the nervous system, thereby targeting both central and peripheral sources of prolonged inflammation. Future studies using labeled hSC-Exos will be required to follow the injected exosomes to verify regional patterns of accumulation after systemic administration.

Exosomes secreted from different phenotypic cell types may carry distinct cargoes that can potentially exert neuroprotective, regenerative, or pathological effects on recipient cells.^31^ In terms of the cargo of rodent SC-Exos, Wei and colleagues^92^ using primary Schwann cells derived from the sciatic nerve of adult Wistar rats reported potentially neurorestorative proteins and signaling pathways involved in axonal regeneration and inflammation inhibition, signal transduction, and cell communication.

An important point is that previous studies have used rodent SC-Exos and not used human cells as a source of the exosomes. There is limited research examining the efficacy of hSC-Exos in neurotrauma or other neurological conditions. In the current study, we utilized hSC derived exosomes to enhance the potential for translating this experimental treatment into the clinic.

An advantage of using exosomes as a delivery system for therapeutic approaches is their ability to potentially target multiple cell types.^93,94^ Previous studies established that PBBI increases the number of activated microglia at both acute and more chronic time points after injury.^40,53^

In this study, we report that delivery of hSC-Exos 30 min after PBBI significantly increased the number of resting and primed microglia while decreasing the number of activated microglia at two days after injury. At three weeks after injury, the administration of hSC-Exos also significantly reduced overall contusion volume compared with vehicle treated PBBI animals and decreased the number of amoeboid microglia in perilesional areas. These results indicate that a single dose of hSC-Exos early after pTBI can influence microglia phenotypic responses in both the acute and chronic phases after injury.

It is well known that microglial activation plays an important role in the neuroinflammatory response after TBI.^53,95^ As part of the innate immune response to acute and progressive brain injury, microglia are activated by damage associated molecular patterns (DAMPs) and release pro-inflammatory mediators such as interleukin (IL)-1β.^96^ The inflammasome is a component of the innate immune system and implicated in the inflammatory response after TBI and other neurological conditions.^10,97–99^

This multi-protein complex plays a role in the activation of caspase-1 and the processing of IL-1β and IL-18.^100–102^ The activation of caspase-1 leads to a regulated form of cell death, through cleavage of gasdermin-D (GSDMD), known as pyroptosis.^103^ Pyroptosis is characterized by a pore formation in the plasma membrane leading to the rupture of the membrane and the release of the intracellular contents including extracellular IL-1β and IL-18.^104^

Previous studies from our group have shown that PBBI induces microglial caspase-1 activation and pyroptosis 48h after PBBI and that this is inhibited by treatment with an inflammasome inhibitor, anti-ASC.^40^

In the present study, we demonstrate that hSC-Exos treatment also decreases caspase-1 activation in activated microglia in the acute phase after PBBI. We chose to study an acute time point for our flow cytometry studies based on our previous findings showing increased caspase-1 activation in activated microglia at 48h post-PBBI.^53^ This is the first study to examine hSC-Exos as a therapeutic strategy for TBI demonstrating that the inflammasome signaling pathway is involved in this therapeutic mechanism.

In a recent TBI study, Tang and coworkers^105^ reported that adipose-derived stem cell exosomes reduced brain injury through a NLRP3 signaling pathway associated with inflammasome activation. Further TBI studies are necessary to clarify the mechanism of caspase-1 inhibition after hSC-Exos. Because our previous studies demonstrated that the NLRP3 inflammasome is activated 48h after PBBI, it will be important to examine NLRP3 expression after hSC-Exos treatment in the acute phase post-PBBI.

In addition to examining levels of caspase-1 expression in activated microglia, we also examined caspase-1 expression and pyroptotic cell death in all live cells. We found a non-significant reduction of caspase-1 activation with hSC-Exos treatment. These current findings therefore suggest that hSC-Exos may reduce caspase-1 activation through a more targeted cell specific mechanism, via the microglial response.

Flow cytometric results from our previous studies in the PBBI model reported that inhibition of the inflammasome using an anti-ASC antibody (IC100) reduced the number of CD11+ leukocytes undergoing caspase-1 activation pyroptosis.^40^ To compare our current findings with our previous work, we also examined CD11b and CD45 positive cells using flow cytometry to differentiate the number of endogenous microglia from the number of infiltrating leukocytes.

As reported previously in the PBBI model as well as other published TBI studies, the number of infiltrating CD11b positive leukocytes was increased 48h after PBBI. We showed no significant change in the number of these cells after the delivery of hSC-Exos. In this severe model of PBBI, the intravenous delivery of hSC-Exos after injury may therefore be primarily reducing the inflammatory response by regulating macrophage/microglia polarization at the 48h time point. ^106^

This study supports previous findings and suggests the possibility that hSC-Exos can decrease neuroinflammation and enhance neurological repair by the reduction of microglial inflammasome activation. Although the model used in this study mimics a penetrating TBI, there are implications that hSC-Exos treatment may have beneficial effects in other forms of TBI. Heterogeneity in the TBI population is a significant challenge when planning clinical trials studying therapeutic interventions. The PBBI model is a rather severe TBI model and additional studies using other TBI models with different injury severities would help validate this human exosome-based treatment and enhance the translation of this experimental treatment.

The current study shows that hSC-Exos reduced the number of activated microglia at early time points and amoeboid appearing microglia at later time points. This is important because there are limited treatment strategies for TBI that target both the acute and more chronic phases after injury. Additional studies examining dose responses and the therapeutic window for hSC-Exos treatment after TBI will also be important in determining whether delaying treatment using clinically relevant treatment windows also has benefits in promoting behavioral outcomes.

In this initial study to evaluate the effects of hSC-Exos in a TBI model, only male rats were used. Because sex is an independent variable in the pathophysiology and management of TBI, future studies using both males and females are required to evaluate the potential of sex-dependent effects of hSC-Exos on the inflammatory response after PBBI.^107^ Importantly, additional studies are in progress to determine the cargo of hSC-Exos because only rodent SC exosomes have been analyzed.

## Conclusion

We examined the effects of hSC-Exos treatment in an established model of pTBI. We report that treatment with hSC-Exos 30 min after PBBI (a) reduced the number of activated microglia 48h post-injury, (b) reduced the inflammasome protein caspase-1 expression in activated microglia at 48h post-injury, (c) decreased lesion volume at 21 days post-injury, and (d) increased the number of resting and primed microglia while decreasing the number of amoeboid microglia at 21 days post-injury.

The current study provides the first evidence for hSC-Exos as a novel therapeutic intervention for TBI by reducing microglial activation, attenuating the innate immune response to trauma, and significantly reducing contusion volume in a severe TBI model. These findings indicate that the systemic delivery of hSC-Exos early after PBBI has anti-inflammatory effects that contributes to neurological protection and improved structural integrity. Because hSC and hSC-Exos have been used in clinical settings for other indications, the potential clinical translation of this experimental therapeutic treatment for TBI is possible if beneficial results can also be replicated in other TBI models.

## Transparency, Rigor, and Reproducibility Summary

This manuscript is designated as a translational therapeutic study as it involves non-human animal subjects with characteristics relevant to human traumatic brain injury. This study was not formally registered as the proposal describing the work was reviewed extensively by multiple committees and updated since 2016. The proposal received funding by the United States Department of Defense, W81XWH-16-2-0008 and the knowledge was in the public domain. Funding was also received by NIH/NINDS 1R37NS133195 and 1RF1NS125578. The analysis plan was not formally pre-registered, Drs. Dietrich and Bramlett, as team members with primary responsibility for the analysis certify that the analysis plan was pre-specified prior to initiation of the study. A power analysis based on pilot data and previous publications was used to set the desired effect size at 0.7. A sample size of N = 5-6 for the flow cytometry and N = 10 for histopathology outcome was calculated using G*Power3.1 (Power set at 0.80 and alpha at 0.05). The investigators were blinded to the experimental groups for all data analysis. The hSC-Exos cell dose was based on previous work in the laboratory. All materials required to perform the study are available from commercial sources and hSC-Exos were obtained from the University of Miami Interdisciplinary Stem Cell Institute using Good Manufacturing Practices. The experimental injury model is an established standard in the field. The sample sizes reflect the number of independent measurements and are comparable to previous reports with the model. Correction for multiple comparisons was performed using GraphPad Prism. Data and analytic code from this study are available from the corresponding author. Materials used to conduct the study are not publicly available.
